# Skin layer classification by feedforward neural network in bioelectrical impedance spectroscopy

**DOI:** 10.2478/joeb-2023-0004

**Published:** 2023-08-10

**Authors:** Kiagus Aufa Ibrahim, Marlin Ramadhan Baidillah, Ridwan Wicaksono, Masahiro Takei

**Affiliations:** 1. Department of Mechanical Engineering, Graduate School of Science and Engineering, Chiba University, Chiba, Japan; 2. Research Center for Electronics, National Research and Innovation Agency, KST Samaun Samadikun, Bandung, Indonesia; 3. Electrical and Information Engineering Department, Faculty of Engineering, Universitas Gadjah Mada, Yogyakarta, Indonesia

**Keywords:** Bioelectrical impedance spectroscopy, Conductivity change, Distribution of relaxation times, Feedforward neural network

## Abstract

Conductivity change in skin layers has been classified by source indicator *o^k^* (*k*=1: Stratum corneum, *k*=2: Epidermis, *k*=3: Dermis, *k*=4: Fat, and *k*=5: Stratum corneum + Epidermis) trained from feedforward neural network (FNN) in bioelectrical impedance spectroscopy (BIS). In BIS studies, treating the skin as a bulk, limits the differentiation of conductivity changes in individual skin layers, however skin layer classification using FNN shows promise in accurately categorizing skin layers, which is essential for predicting source indicators *o^k^* and initiating skin dielectric characteristics diagnosis. The *o^k^* is trained by three main conceptual points which are (i) implementing FNN for predicting *k* in conductivity change, (ii) profiling four impedance inputs *α_ξ_* consisting of magnitude input *α*|_*z*_|, phase angle input *α_θ_*, resistance input *α_R_*, and reactance input *α_x_* for filtering nonessential input, and (iii) selecting low and high frequency pair $\left(f_{r}^{lh}\right)$ by distribution of relaxation time (DRT) for eliminating parasitic noise effect. The training data set of FNN is generated to obtain the *α_ξ_* ∈ ***R***^10×17×10^ by 10,200 cases by simulation under configuration and measurement parameters. The trained skin layer classification is validated through experiments with porcine skin under various sodium chloride (NaCl) solutions *C_NaCl_* = {15, 20, 25, 30, 35}[mM] in the dermis layer. FNN successfully classified conductivity change in the dermis layer from experiment with accuracy of 90.6% for the bipolar set-up at $f_{6}^{lh}=10\;\&100\;[\text{kHz]}$ and with the same accuracy for the tetrapolar at $f_{8}^{lh}=35\;\&100\;[\text{kHz]}$. The measurement noise and systematic error in the experimental results are minimized by the proposed method using the feature extraction based on *α_ξ_* at $f_{r}^{lh}$.

## Introduction

Skin is a multi-layer dielectric medium with different electrical properties related to structural characteristics [1], chemical composition variations [2], and different biological functions, in which the assessment of each layer leads to a better understanding of its pathophysiology. As a common modality to measure dielectric characteristics in biological tissue, bioelectrical impedance spectroscopy (BIS) is well-known as low-cost, sensitive to chemical composition, and portable for clinical measurement, which is capable of measuring the electrical conductivity of the human skin layer [3]. BIS for human skin is utilized to assess skin rubor (or erythema) [4], skin cancer [5], diabetes [6], edema [7], skin irritation [8], skin thickness [9], and drug delivery [10]. Each skin layer contributes differently, allowing current to flow during measurement. Thus, any conductivity change in each skin layer affects the measured impedance. However, most BIS studies considered skin as a bulk instead of having layer composition. In the case of the conductivity change occuring at a particular skin layer, the traditional BIS shows the impedance change but cannot determine which skin layer has changed its conductivity because the equivalent circuits of each layer are connected and behave as a parallel circuit. Consequently, BIS is not able to solve the indistinguishability of skin layers in conductivity change.

In order to solve the problem, skin layer classification by machine learning algorithm (MLA) is introduced. MLA have been proposed by some studies such as Natasha et al. to detect breast cancer [11], Gessert et al. to detect melanoma [12], and Kawahara et al. to detect skin lesions [13]. The studies of tissue disorders based on BIS and MLA illustrate the ability of MLA skin layer classification. For impedance measurements, Feedforward Neural Network (FNN) is appropriate because it can model complex relationships between inputs and outputs, making it a powerful tool for high-accuracy classification tasks that can be performed quickly with less computational power and can use parallel computation to increase the algorithm’s efficiency [14]. The skin layer classification based on impedance measurement, which focuses specifically on predicting source indicator *o^k^*, is a preliminary step to skin dielectric characteristics diagnosis. FNN is employed for predicting source indicator *o^k^*.

As a post-processing BIS for predicting conductivity change in time domain, the distribution of relaxation times (DRT) [15] was proposed. By the principle of superposition, the impedance can be represented as a series connection of an infinite number of electrical equivalent circuit (EEC) made of a parallel connection of a resistive polarization part and a capacitive part [16]. DRT assumes that the measured impedance is an infinite number of RC elements with continuous relaxation time [17]. However, DRT requires knowledge of baseline value of the conductivity change on a specific time constant. Any conductivity changes with frequency-dependent behaviour affect the amplitude of the relaxation time function, which is not limited to one time constant. Thus, DRT is adequately used whenever the unwanted conductivity change can be detected and eliminated. The baseline value with a known conductivity value prior to the impedance measurement is needed to classify the skin conditions in the time domain, which is related to a conductivity change in a specific skin layer. Otherwise, the conductivity change cannot be classified. To overcome this challenge, four impedance inputs *α_ξ_* with time-difference analysis are introduced.

In order to overcome the baseline value problem and to determine the desired skin layer accurately, the impedance changes of BIS are transformed into four impedance inputs *α_ξ_* of FNN. They are presented with time-difference analysis, which are the magnitude input (MX) *α_|Z|_*, phase angle input (PX) *α_θ_*, resistance input (RX) *α_R_*, and reactance input (IX) *α_X_*. The MX, PX, RX and IX have been proposed by Ollmar et al. without considering the time difference analysis in order to diagnose the oral mucosa which are chosen solely based on physically fundamental aspects of the behavior of skin in the lower frequency and higher frequency [18]. Additionally, to increase the relation of training data features with the FNN results, two possible injection patterns *φ_s_*, which are Bipolar and Tetrapolar, are presented and compared with each other. The two injection patterns *φ_s_* have different performances in extracting the electrical properties of the viable tissues because the electrode-length-to-skin-thickness ratio *δ_b_* significantly affects the electrical current penetration depth.

Conductivity change related to physiological change is always affected by parasitic noise, such as variation of temperature or moisture content in the tissues. Hence, the parasitic noise effect on impedance measurements can be eliminated by selecting the frequency pair whose effect is negligible. In this regard, DRT is employed to select the frequency pair where the conductivity changes from the skin layer more dominantly than other conductivity sources.

Therefore, we come up with three main conceptual points, which are (i) FNN implementation for predicting *o^k^* in conductivity change, (ii) four impedance inputs *α_ξ_* profiles consisting of magnitude input *α_|z|_*, phase angle input *α_θ_*, resistance input *α_R_*, and reactance input *α_X_*, and (iii) frequency pair $f_{r}^{lh}$ selection by the distribution of relaxation time (DRT) in order to accurately predict the conductivity change in the skin layer. In summary, Appendix A shows the comparison with the related literature studies to highlight the contribution of this study.

This paper presents a skin layer classification method employing FNN based on the conductivity change measured by bioelectrical impedance spectroscopy. In this study, three objectives are presented, which are (1) The validation accuracy of source indicator *o^k^* trained from FNN, (2) The validation accuracy based on experiment, and (3) Impedance inputs *α_ξ_* evaluation in performing feature extraction.

## Skin layers classification of conductivity change in skin layers by source indicator trained from FNN

### *Implementing FNN for predicting* k *in conductivity change*

**Fig. 1:**
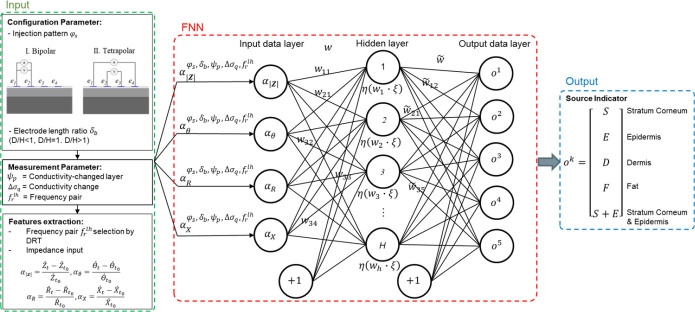
Schematic flow of skin layer classification of conductivity change

**Fig. 1** shows the schematic flow of FNN. FNN is a fully connected layer of the neural network and connections from the network inputs to each subsequent layer from the previous layer, known as multi-layer perceptron (MLP). A structure of FNN consists of one hidden layer with 100 neurons, the four impedance inputs *α_ξ_* in the input layer, and the five source indicators *o^k^* in the output layer. This fully connected FNN structure has (*ξ*×*u*)+(*u*×*o*) total number of weight connections, where *ξ* = {1, …, Ξ} is data input in the input layer, *u* = {1, …, *U*} is neuron in the hidden layer, and *o* = {1, …, *O*} is the number of data output in the output layer. The weight connections are used in order to perform the computation. The computation steps of the network of one hidden layer for *n*th sample in training data set is as follows:

1) The first step is summing the weights in the hidden layer:
1 $$S_{u}^{n}=\sum_{1}^{U}w_{u\xi }\alpha_{\xi }+\beta \xi$$
  where *α_ξ_* is the input data (which are impedance inputs), *w_uξ_* is the weight vector connecting the input neurons *ξ*th and the hidden layer neuron *u*th, and *β_ξ_* is the input variable’s bias term,
2) The second step is feeding the summations of the first step NN computation to the neurons’ output using the rectified linear unit (ReLU) activation function *g_u_*:
2 $$g_{u}\left(S_{u}^{n}\right)=\{\begin{array}{ll} S_{u}^{n}, & S_{u}^{n}>0\\ 0, & S_{u}^{n}\leq 0 \end{array}$$
  where $S_{u}^{n}$ is the sum of weights,
3) The last step is calculating the output neurons:
3 $$y_{o}^{n}=\overset{U}{\underset{1}{\sum }}{\tilde{w}}_{uo}g_{u}+\beta_{u}$$
  where $y_{u}^{n}$ is the output of hidden layer neuron *u*th, ${\tilde{w}}_{uo}$ is the weight between the output variable $y_{o}^{n}$ and hidden layer neuron *u*th, *β_u_* is the output variable’s bias term, *n* is the number of samples which is *F* (number of frequency pair of lower frequency and higher frequency) times *t* (number of impedance measurements in a time domain), *F* = {1, …, *Γ*}, and *t* ={1, … *T*}.

The training process based on the supervised learning employs a tuning process to control the weight (*w_uξ_* and ${\tilde{w}}_{uo}$) and bias (*β_ξ_* and *β_u_*) parameters based on the minimizing of error rate including both skin layer classification and approximation errors.

### Profiling four impedance inputs *α_ξ_*

In order to minimize the unnecessary training data of FNN, this study introduces impedance inputs *α_ξ_* as a data feature extraction which are the magnitude input (MX) *α_|z|_*, phase angle input (PX) *α_θ_*, resistance input (RX) *α_R_*, and reactance input (IX) *α_x_*. Considering that the skin layer classification of conductivity change is in the time domain. Thus, the impedance inputs *α_ξ_* in the time domain are as the following equations:

4 $$\alpha_{\left|Z\right|}=\frac{{\overset{´}{Z}}_{t}-{\overset{´}{Z}}_{t_{o}}}{{\overset{´}{Z}}_{t}{}_{{}_{o}}}$$

5 $$\alpha_{\theta }=\frac{{\overset{´}{\theta }}_{t}-{\overset{´}{\theta }}_{t_{o}}}{{\overset{´}{\theta }}_{t}{}_{{}_{o}}}$$

6 $$\alpha_{R}=\frac{{R^{\prime }}_{t}-{R^{\prime }}_{t_{\text{o}}}}{{R^{\prime }}_{t}{}_{{}_{\text{o}}}}$$

7 $$\alpha_{X}=\frac{{\overset{´}{X}}_{t}-{\overset{´}{X}}_{t_{o}}}{{\overset{´}{X}}_{t}{}_{{}_{o}}}$$

where

8 $$\overset{´}{Z}=\frac{\text{abs}\left(Z_{l}\right)}{\text{abs}\left(Z_{h}\right)}$$

9 $$\overset{´}{\theta }=\arg\left(Z_{l}\right)-\arg\left(Z_{h}\right)$$

10 $$\overset{´}{R}=\frac{\text{re}\left(Z_{l}\right)}{\text{abs}\left(Z_{h}\right)}$$

11 $$\overset{´}{X}=\frac{\text{im}\left(Z_{l}\right)}{\text{abs}\left(Z_{h}\right)}$$

where *t* is an arbitrary amount of time after a reference time *t*_0_, *abs*(*Z*) is the magnitude (modulus) of the complex electrical impedance, *arg*(*Z*) is the argument (phase angle) in degrees, *re*(*Z*) is the resistance of the complex electrical impedance (*re*(*Z*) = *abs*(*Z*) * *cos* [*arg*(*Z*)]), and *im*(*Z*) is the reactance of the complex electrical impedance (*im*(*Z*) = *abs*(*Z*) * *sin* [*arg*(*Z*)]). *Z_l_* and *Z_h_* represent the impedance at low and high frequencies, respectively, where the frequencies are based on the frequency pair $f_{r}^{lh}$ selection.

In fact, any change in conductivity *σ* and permittivity *ε_r_* of the skin layer affects all *α_ξ_*. The *Ŕ* reflects conductivity changes; the $\overset{´}{X}$ reflects mainly reactance changes, which are of capacitive nature; the *Ź* reflects changes along the length of the vector describing the impedance in complex space, which is emphasized if the real and reactance change are in the same direction and proportion; the $\overset{´}{\theta }$ is emphasized if the real and reactance change are in different directions and different proportions. All *α_ξ_* reside solely on physically fundamental aspects of the behavior of electrical impedance in the selected frequency range [7]. Thus, the accuracy of equations (4) to (7) depends on the frequency pair $f_{r}^{lh}$ selection. The DRT is used to select the frequency pair based on the time-constant domain, which is related with the skin layer as explained in the next subsection.

### Selecting frequency pair $f_{r}^{lh}$ by distribution of relaxation times (DRT)

The predicted impedance of DRT *Z_drt_* [Ω] is represented by the following equation:

12 $$Z_{drt}\left(f\right)=R_{\infty }+\int_{0}^{\infty }\frac{\gamma \left(\ln\;\tau \right)}{1+j2\pi f\tau }\text{d}\left(\ln\;\tau \right)$$

where *R*_∞_[Ω] is resistance at the high frequency, *γ*(ln *τ*)[Ω] is the distribution function of the total polarization resistance along the relaxation time *τ*[*s*], and $\int_{-\infty }^{\infty }\gamma \left(\ln\;\tau \right)\text{d}\left(\ln\;\tau \right)=R_{p}$ is a normalized function over the entire relaxation time τ where *R_p_* is the polarization resistance.

In order to retrieve the distribution of *γ*(ln *τ*), an approximation by the sum of *M* radial basis function (RBFs) is used. Hence, equation (12) can be expressed as follows [17]:

13 $$Z_{\text{drt}}(f;\Theta )=R_{\infty }+\sum_{m=1}^{M}\Theta_{m}\int_{0}^{\infty }\frac{g_{m}(\ln\;\tau )}{1+j2\pi f\tau }\text{d}(\ln\;\tau )$$

where, *g_m_*(ln *τ*) ≜ ^*e*−(*μ*(|ln*τ*−ln*τ_m_*|)^2^^ [Ω] is the RBFs with each function is center at the relaxation time *τ_m_* and a full width at half maximum (FWHM) as *μ*. While ***Θ*** = [*Θ*_1_, ⋯, *Θ_m_*]^*T*^ is a vector parameter related to the amplitude of RBFs.

Calculating the *γ*(ln *τ*) in equation (13) is an ill-posed problem case. Currently, there are several available methods to overcome the DRT ill-posed problem: an evolutionary programming method proposed by Oz et al. [19], a monte-carlo method proposed by Tuncer and McDonald [20], a discrete Fourier transformation proposed by Schichlein et al. [21] and a fitting approach proposed by Sonn et al. [22]. Using a discrete Fourier transformation is not trivial because of requiring an indispensable data exploration and filtering of high-quality impedance data [23]. Alternatively, a fitting approach for DRT is proposed based on fitting the resistance of the spectra to a number of RC elements with fixed time constants [22]. A further improvement by the Ciucci research group for the fitting approach is employing a Tikhonov regularization [16]. In this study, a mathematical toolbox called the DRTtools toolbox proposed by Wan et al. is used. The main goal is to fit the data between the impedance *Z_exp_* [Ω] from experiment against with the model *Z_drt_* by optimization of *γ*(ln *τ*) [16]. The optimization of *γ*(ln *τ*) is retrieved as the optimal parameters vector $\Theta *=\underset{\Theta :\Theta \geq 0}{\text{argmin}}\;V\left(\Theta \right)$ by minimizing the error least square between the measured impedance and the predicted impedance as follows:

14 $$V(\Theta )=\|((R_{\infty }1)+A^{\text{re}}\Theta )-Z_{\exp}^{\text{re}}{\|}_{2}^{2}+\|\left(A^{\text{im}}\Theta \right)-Z_{\exp}^{\text{im}}{\|}_{{}^{2}}^{2}+\lambda \|\Theta {\|}_{2}^{2}$$

where *L* is the number of frequency points, *λ* > 0 is the hyperparameters to tune the error computation, and ${\text{A}}^{\text{re}}\in {\mathbb{R}}^{L\times M}$ and ${\text{A}}^{\text{im}}\in {\mathbb{R}}^{L\times M}$ are the matrix notion of parameters obtained from the real $Z_{drt}^{re}$ and reactance $Z_{drt}^{im}$ of the predicted impedance *Z_drt_*, which are expressed as follows:

15 $$Z_{drt}^{re}=R_{\infty }+\overset{M}{\underset{m=1}{\sum }}\Theta_{m}\left[\underset{A^{\text{re}}}{\underset{︸}{\overset{\infty }{\underset{0}{\int }}\frac{g_{m}(\ln\;\tau )}{1+{\left(2\pi f\tau \right)}^{2}}\text{d}(\ln\;\tau )}}\right]$$

16 $$Z_{drt}^{im}=-\sum_{m=1}^{M}\Theta_{m}\left[\underset{A^{\text{im}}}{\underset{︸}{\int_{0}^{\infty }\frac{2\pi f\tau g_{m}\left(\ln\;\tau \right)}{1+{\left(2\pi f\tau \right)}^{2}}\text{d}\left(\ln\;\tau \right)}}\right]$$

Once the *γ*(ln *τ*) is retrieved, it provides a plot of *γ*(ln *τ*) against *τ.* Hence, different relaxation mechanisms can be easily identified as a representation of frequency information where the conductivity change influences significantly or insignificantly on the impedance *Z*. Some of the frequency pairs are selected heuristically in order to calculate the impedance inputs *α_ξ_* as shown in equations (4) to (7).

## Generation of training data set by numerical simulation

### Method and condition of numerical simulation

Numerical simulation studies are employed using finite element method (FEM) software to generate input for training data sets. The simulation of electric potential *ϕ*(**r**) inside a subdomain Ω is produced by placing a current across the surface skin in the boundary *∂*Ω on each electrode transmitter with the injected current *i* [24].

**Fig. 2:**
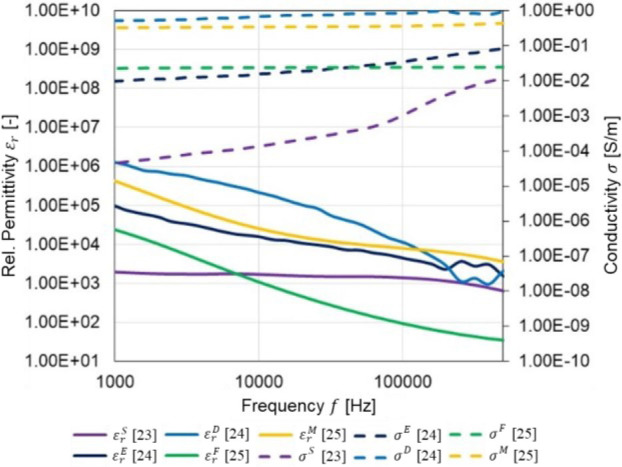
Electrical properties of skin, fat, and muscle.

17 ∇⋅(σ*(r))∇ϕ(r)=0, r∈Ω

18 $$\phi \left(r\right)+Z_{c}\sigma *\left(r\right)\frac{\partial \phi \left(r\right)}{\partial n}=U_{l},\;r\in e_{l},\;l=\left\{1,\;\ldots ,\;L\right\}$$

19 $$\int_{\partial \Omega^{e_{l}}}\sigma *(r)\frac{\partial \phi (r)}{\partial n}dS=I,\;r\in \partial \Omega^{e_{l}}$$

20 $$\sigma *(r)\frac{\partial \phi (r)}{\partial n}=0,\;r\in \partial \Omega \backslash \overset{L}{\underset{l=1}{\cup }}e_{l}$$

where $\sigma *:=\sigma +2\pi f\varepsilon \in \mathbb{C}\left[\text{S}\;{\text{m}}^{-1}\right]$ is the nonhomogenous admittivity property of skin layer, *σ* and *ε* are the conductivity and absolute permittivity [F m^−1^] respectively in Ω at the frequency *f*, ϕ(r)∈ℂ[V] is the electric potential distribution, and **r** := (*x, y, z*) is the coordinate system in subdomain Ω. **Table 1** shows the geometry of the skin layer model with BIS electrodes *e_l_*. The contact impedance on each electrode is *Z_c_* = 50 [Ω]. Meanwhile, the electrical properties of stratum corneum [25], epidermis, dermis [26], and fat [27] are adopted from several literatures. **Fig. 2** shows the comparison of conductivity σ(**r**) and relative permittivity ε_*r*_(**r**) of each skin layer.

**Table 1:** Parameters for training data set

Configuration parameter		Measurement parameter		
Injection pattern *φ_s_*	Electrode-length-to-skin-thickness ratio *δ_b_*	Conductivity-changed layer *ψ_p_*	Conductivity change Δ*σ_q_* [%]	Frequency pair $f_{r}^{lh}$ [kHz]
I. Bipolar **Figure:** 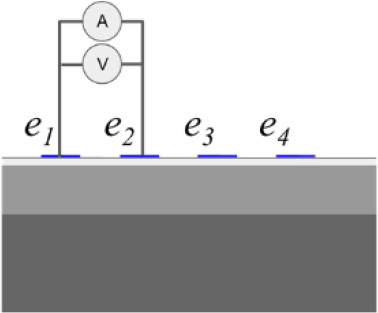 II. Tetrapolar **Figure:** 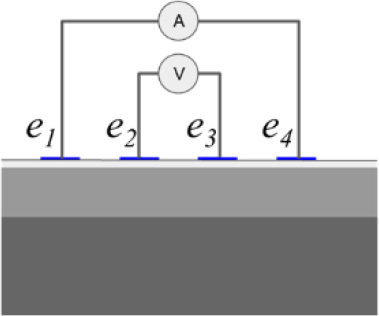	*D/H* < 1, *D/H* = 1, *D/H* > 1 **Figure:** 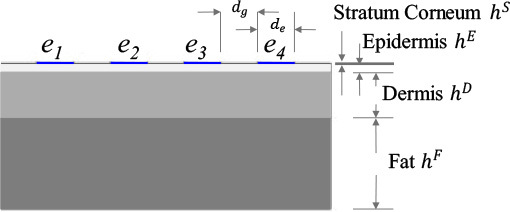 Fixed electrode gap: *d_g_* = 1 [mm] Variable electrode diameter: *d_e_* = {1, 2, 3}[mm] Electrode length: *D* = *d_g_* + *d_e_* Fixed each layer thickness: *h^S^* = 50 [μm], *h^E^* = 0.45 [mm], *h^D^* = 2.5 [mm], *h^F^* = 5 [mm] Fixed skin thickness: *H* = *h^S^* + *h^E^* + *h^D^* + *h^F^*	*ψ*_1_ = Stratum Corneum only (S) *ψ*_2_ = Epidermis (E) only *ψ*_3_ = Dermis (D) only *ψ*_4_ = Fat (F) only *ψ*_5_ = S + E *ψ*_6_ = E + D *ψ*_7_ = D + F *ψ*_8_ = S + E + D *ψ*_9_ = E + D + F *ψ*_10_ = S + E + D + F	Δσ_1_ = −20 Δσ_2_ = −17.5 Δσ_3_ = −15 Δσ_4_ = −12.5 Δσ_5_ = −10 Δσ_6_ = −7.5 Δσ_7_ = −5 Δσ_8_ = −2.5 Δσ_9_ = 0 Δσ_10_ = 2.5 Δσ_11_ = 5 Δσ_12_ = 7.5 Δσ_13_ = 10 Δσ_14_ = 12.5 Δσ_15_ = 15 Δσ_16_ = 17.5 Δσ_17_ = 20	$\begin{array}{l} f_{1}^{lh}=2\&10\\ f_{2}^{lh}=2\&35\\ f_{3}^{lh}=2\&100\\ f_{4}^{lh}=2\&225\\ f_{5}^{lh}=10\&35\\ f_{6}^{lh}=10\&100\\ f_{7}^{lh}=10\&225\\ f_{8}^{lh}=35\&100\\ f_{9}^{lh}=35\&225\\ f_{10}^{lh}=100\&225 \end{array}$

1 Δσ_9_ = 0 [%] is considered as standard. For each conductivity-changed layer, the other layer(s) are fixed with standard conductivity change value. Case number follow this rule: $\varphi_{s\_}\delta_{b\_}\psi_{p\_}\Delta \sigma_{q\_}f_{r}^{lh}$ (for example: I_D/H < 1_S_-20_2&10 which refers to injection pattern: *φ_I_* = Bipolar_*δ_1_ = *D/H* < 1, *ψ*_1_* = *S*, Δ*σ*_1_ = −20, and $f_{3}^{lh}=2\&100$) Total case: $2\left(\varphi_{s}\right)\times 3\left(\delta_{b}\right)\times 10\left(\psi_{p}\right)\times 17\left(\Delta \sigma_{q}\right)\times 10\left(f_{r}^{lh}\right)=10200$

### Input data for machine learning

#### 1) Variation from standard conductivity change

**Table 1** shows the conditions of the training data set generated by simulation. **Fig. 2** shows the variation of conductivity change Δ*σ_q_* is defined from -20% to +20% from the conductivity *σ* value. The relative permittivity *ε_r_* value is assumed constants for all the cases. The impedance inputs *α_ξ_* are calculated from the impedance measurement at different frequency ranges *f* ={2, 10, 35, 100, 225}[kHz] selected through DRT from simulation and experiment data in the prior study. From this frequency range, the frequency pair $f_{r}^{lh}$ in equations (8) to (11) are selected, as shown in **Table 1**.

#### 2) Electrode Length to Skin Thickness

The electrode length *D*, which combines the electrode diameter *d_e_* and the gap between the electrodes *d_g_*, plays an important role for the current penetration depth. In order to obtain a deeper electrical current penetration, *D* should be larger compared to the skin thickness *H*. As the electrical current penetration depth gets deeper, more tissue layers can be validated through BIS. Selecting the proper electrode-length-to-skin-thickness ratio *Δ_b_* is difficult because skin thickness is unknown during the BIS measurement. Consequently, the skin layer classification of conductivity change should consider the effect of *D/H*. More complicated variation of the electrode length is analyzed. However, the study is simplified by the three different conditions, i.e., *d_e_* ={1,2,3} [mm]. Also, *H* is constant as shown in **Table 1**, which includes the thickness of stratum corneum *h^s^*, epidermis *h^E^*, dermis *h^D^*, and fat *h^F^* layer. Thus, there are three different conditions of electrode-length-to-skin-thickness ratio *δ_b_*: *D/H*< 1, *D/H* = 1, and *D/H* > 1.

#### 3) Injection Patterns

In addition to the conductivity changes, evaluating different injection patterns is also considered in order to obtain the sensitivity of each injection pattern in the skin layer classification of conductivity change. **Table 1** shows a single bioimpedance measurement based on a colinear array that employs two injection patterns *φ_s_* called bipolar and tetrapolar. Although, the tetrapolar configuration is commonly used as injection patterns to obtain the conductivity change information based on its capability to reduce an electrode-skin contact impedance. However, there is a trade-off between the electrode array area and the sensitivity to detect the conductivity change information according to its current pathway. Accordingly, other injection patterns are still adapted in some applications.

The bipolar configuration uses two electrodes, transmitting the current and receiving the voltage. The current density under the two electrodes is non-uniform, which results in non-linear impedance measurement to the variance of conductivity changes in the tissues. The impedance measurement of bipolar configuration is a superposition of three impedance sources: the wires impedance, the electrode-skin contact impedance, and the tissue impedance. The tetrapolar configuration uses four electrodes, two electrodes for transmitting the current and another two electrodes for receiving the voltage.

#### 4) Labeling

In the supervised learning FNN, labeling refers to the process of assigning a label or category to input data based on the output of the neural network. In this case, each input data is assigned a case number representing the labeling for each injection pattern on the training data set. According to **Table 1**, the total case number equals 10200 data generated for the training data set. The classification into five groups (S, E, D, F, S+E) is based on the combination of possible conductivity changes in the skin layer. So, the only possible conductivity changes that occurred are S, E, D, F, S+E, and the adjacent layers. Because the objective is to distinguish specific data from particular layers, not layer combinations, considering layer combinations will lead to treating bulk skin, which differs from the goal. In addition, preliminary studies have shown that the conductivity change of the dermis layer hinders the conductivity change of other layers. As a result, layer combinations containing the dermis are treated the same as the class dermis. Considering all conditions, the arrangement of balanced datasets is maintained to achieve proper classification.

### Feedforward Neural Network framework

The FNN in this paper was used as a supervised skin layer classification learner. Feedforward neural network (FNN) in this paper were implemented using a Matlab Machine Learning and Deep Learning Toolbox (Mathworks, Natick, MA, United States) on a laptop with CPU AMD Ryzen 7 PRO 4750U @1.7 GHz. K-fold = 5 is used to validate the accuracy. The accuracy *Acc* [%] is expressed as follows:

21 $$Acc=\frac{T_{\text{predict}}}{T_{\text{samples}}}\times 100$$

where, *T*_predict_ is the number of true predictions and *T*_samples_ is the number total samples used in the training data.

## Training data set by numerical simulation

### Impedance by numerical simulation

**Fig. 3:**
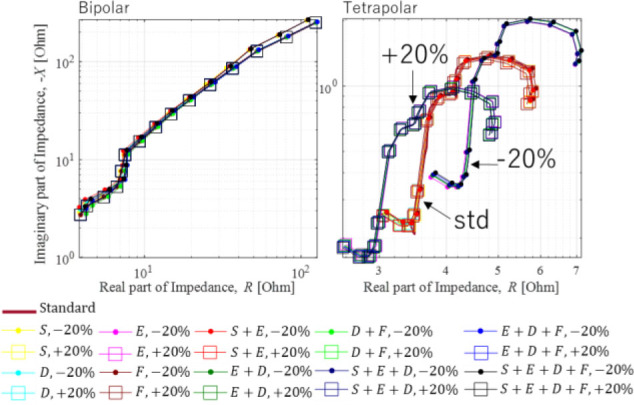
Nyquist plot based on numerical simulation of *φ_s_* in the Δ*σ* and *D/H* < 1 in all frequency range.

**Fig. 3** shows the comparison of the Nyquist plot from the impedance measurement of two *φ_s_* in the case of all ψ_p_ with Δσ_q_ = {−20,0(standard),+20}[%] where D/H < 1 in all frequencies. The impedance spectrums in the Nyquist plot change slightly among the different conductivity change variations. The tendency of the impedance spectrum from the bipolar set-up is similar to the behavior with a Warburg impedance. Meanwhile, in the case of the tetrapolar set-up, the impedance spectrum has some electrical behaviors, which are a charge transfer, dielectric interphase, conductive part, and diffusion part. Nevertheless, the conductivity change is difficult to classify from these data representations.

### Distribution of relaxation times

**Fig. 4:**
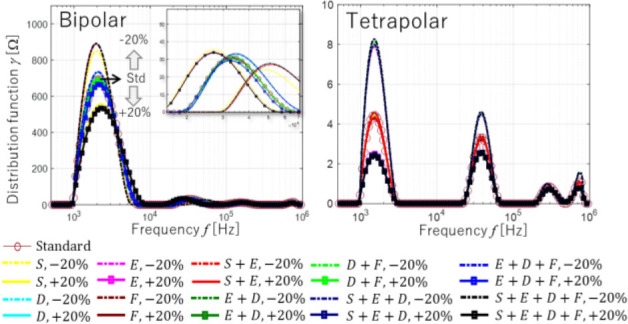
DRT results based on simulation of *φ_s_* in the case of *D/H* < 1 in all frequency range.

The slight changes due to different conductivity shown in the Nyquist plot from simulation is well translated by DRT as shown in **Fig. 4**, which displays multiple local maxima towards higher gamma values as the conductivity increases toward different skin layer acquired from two φ_s_ in the case of all ψ_p_ with Δσ_q_ = {−20,0(standard),+20}[%] where D/H < 1 in all frequencies. By inspection, the DRT serves as an indicator given the retrieved relaxation times of each skin layer. The local maxima observed begin and end by local minimum. Hence, this local minimum between the local maxima is important as indication of the frequency range applied to a skin layer. This observation forms the basis for determining the frequency pairs of each skin layer where changes in conductivity have significant or insignificant effects.

### Feature extraction based on impedance inputs *α_ξ_*

As part of pre-processing data for FNN, feature extraction is performed to select only the features that would contribute most to the quality of data features that will be highly correlated with the output. The introduction of four impedance inputs with a wide range of frequencies leads to feature extraction with several options called one matrix, two matrices, three matrices, and four matrices profile, as shown in **Table 2**.

**Table 2:** Matrix profile of *α_ξ_*

Matrix Profile	Number of input features *α_ξ_*	Details
One matrix	4	$\overset{´}{Z},\overset{´}{\theta },\overset{´}{R},\overset{´}{X}$
Two matrices	6	$\overset{´}{Z}+\overset{´}{\theta },\overset{´}{Z}+\overset{´}{R},\overset{´}{Z}+\overset{´}{X},\overset{´}{\theta }+\overset{´}{R},\overset{´}{\theta }+\overset{´}{X},\overset{´}{R}+\overset{´}{X}$
Three matrices	3	$\overset{´}{Z}+\overset{´}{\theta }+\overset{´}{R},\overset{´}{Z}+\overset{´}{\theta }+\overset{´}{X},\overset{´}{\theta }+\overset{´}{R}+\overset{´}{X}$
Four matrices	1	$\overset{´}{Z}+\overset{´}{\theta }+\overset{´}{R}+\overset{´}{X}$

**Fig. 5:**
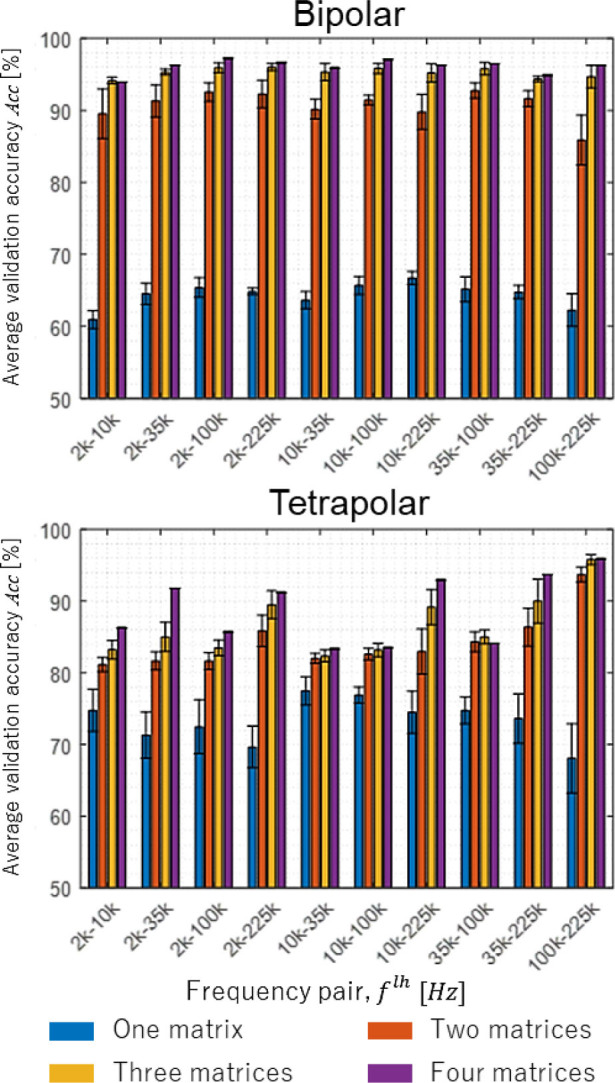
The comparison of FNN results related to feature extraction of *α_ξ_* effect on bipolar and tetrapolar.

**Fig. 6:**
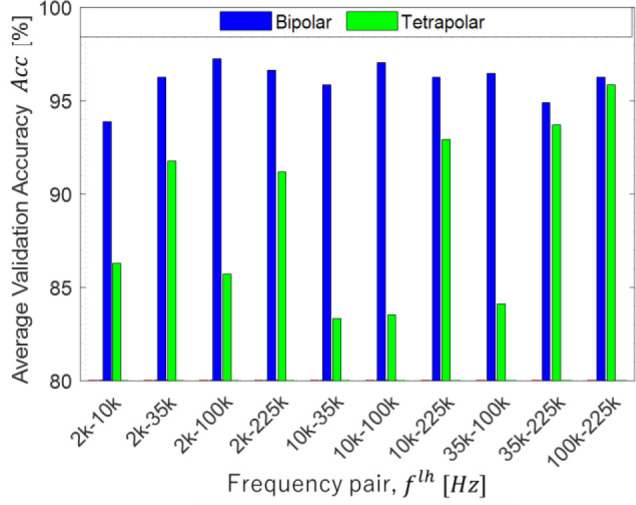
The comparison of FNN results related to feature extraction of *α_ξ_* effect on bipolar and tetrapolar using four matrices profile.

Feature extraction will evaluate the matrices by comparison of average validation accuracy results. **Fig. 5** shows the comparison of FNN average accuracy *Acc* regarding to the matrix of impedance inputs. The figure illustrates that as the number of inputs increases, the accuracy increases for both bipolar and tetrapolar injection patterns. This finding suggests a positive correlation between the number of inputs and accuracy. The four matrices profile shows the highest accuracy in all selected frequency pairs, meaning the selected features on the four matrices profile are relevant to achieve better accuracy on the validation sets, as shown in **Fig. 6**.

## Experiments

### Experimental setup

**Fig. 7:**
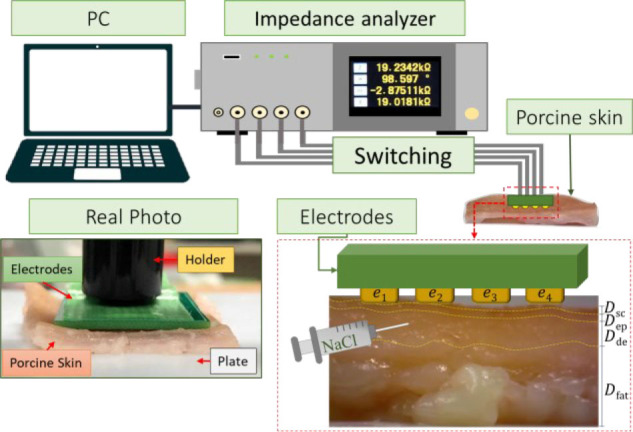
Experimental setup conditions with porcine skin.

**Fig. 7** shows the experimental setup composed of a co-planar sensor with multiple electrodes, an impedance analyzer (IM3570, Hioki EE Corporation, Tokyo, Japan) as a data acquisition system, a switching, and a PC for command and post-processing. The electrodes are manufactured on a printed circuit board and made of stainless steel with a diameter of 1 mm, a height of 1 mm, and distance between both electrodes is 1 mm.

In bipolar mode, one electrode serves as the current injector and high potential electrode in a two-wire measuring system, while the other electrode serves as the ground and low potential electrode. In tetrapolar mode, *e*_1_ acts as the current injector, *e*_2_ serves as the high potential electrode, the low potential electrode is *e*_3_, and the ground electrode is performed by *e*_4_. The ground electrode receives current from the current injector electrode as it passes through the measuring object. The high potential and low potential electrodes simultaneously measure the voltage produced by the current flow.

### Experimental method and conditions

Porcine skin was used as the measurement object because of its comparable mechanical and electrical characteristics to human skin. Several studies have shown that porcine skin is similar to human skin in general structures [28], thickness [29], lipid composition [30], and dielectric properties [31]. While human skin varies from person to person, porcine skin remains a valuable model for human skin studies.

The porcine skin still bound together the muscle and fat. The sample must first be adjusted to the proper temperature (about 35 °C), after which extra layers of fat and muscle must be removed, leaving only the stratum corneum, epidermis, and dermis, along with one layer of fat and one layer of muscle, to maintain accurate measurements throughout the experiment.

In order to validate the proposed method, an experiment with porcine skin is used by creating variation in conductivity by injecting saline water into the dermis layer. Our study divided a large-sized pig skin into five samples based on the NaCl concentration levels. For both the bipolar and tetrapolar injection patterns, each sample underwent three injections at different locations within the dermis layer. Subsequently, we performed three repeated measurements at each location. Therefore, in summary, each sample was subjected to nine measurements. The average of these measurements was then used as the input for the validation dataset in the FNN model, specifically for the dermis (D) layer, as specified in **Table 3**. Saline water, with a concentration of *C_NaCl_* = 25 [mM], is considered the standard value. This experiment’s conditions are to mimic the conductivity change at the dermis layer artificially [32]. The impedance measurement method is bipolar and tetrapolar with *i* = 1 [mA] and frequency pair $f_{r}^{lh}$ as used in the simulation. The electrode-length-to-skin-thickness ratios are *D/H* < 1, *D/H* = 1, and *D/H* > 1.

**Table 3:** Experimental conditions

Configuration parameter		Measurement parameter		
Injection pattern *φ_s_*	Electrode length ratio to skin thickness*δ_b_*	Conductivity-changed layer*y_p_*	Concentration*C_Nacl_* [mM]	Frequency pair $f_{r}^{lh}$ [kHz]
I. Bipolar **Figure:** 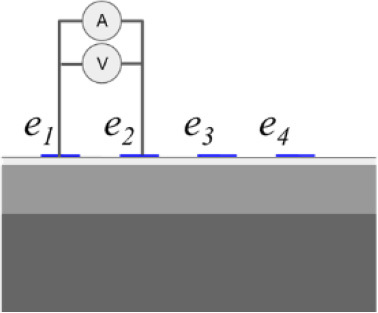 II. Tetrapolar **Figure:** 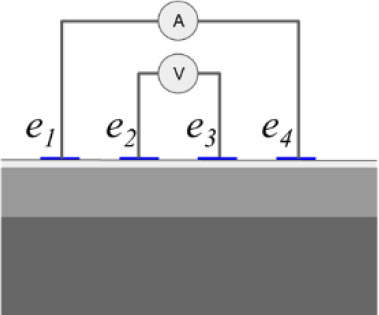	*D/H* < 1, *D/H* = 1, *D/H* > 1 **Figure:** 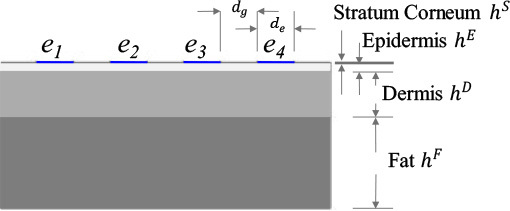	Dermis (D) only	*c*_1_ = 15 *c*_2_ = 20; *c*_3_ = 25; *c*_4_ = 30; *c*_5_ = 35;	$\begin{array}{l} f_{1}^{lh}=2\&10;\\ f_{2}^{lh}=2\&35;\\ f_{3}^{lh}=2\&100;\\ f_{4}^{lh}=2\&225;\\ f_{5}^{lh}=10\&35;\\ f_{6}^{lh}=10\&100;\\ f_{7}^{lh}=10\&225;\\ f_{8}^{lh}=35\&100;\\ f_{9}^{lh}=35\&225;\\ f_{10}^{lh}=100\&225 \end{array}$

### Ethical approval

The research related to animals use has been complied with all the relevant national regulations and institutional policies for the care and use of animals.

## Results and discussion

### Frequency pair $f_{r}^{lh}$ selection by DRT

**Fig. 8:**
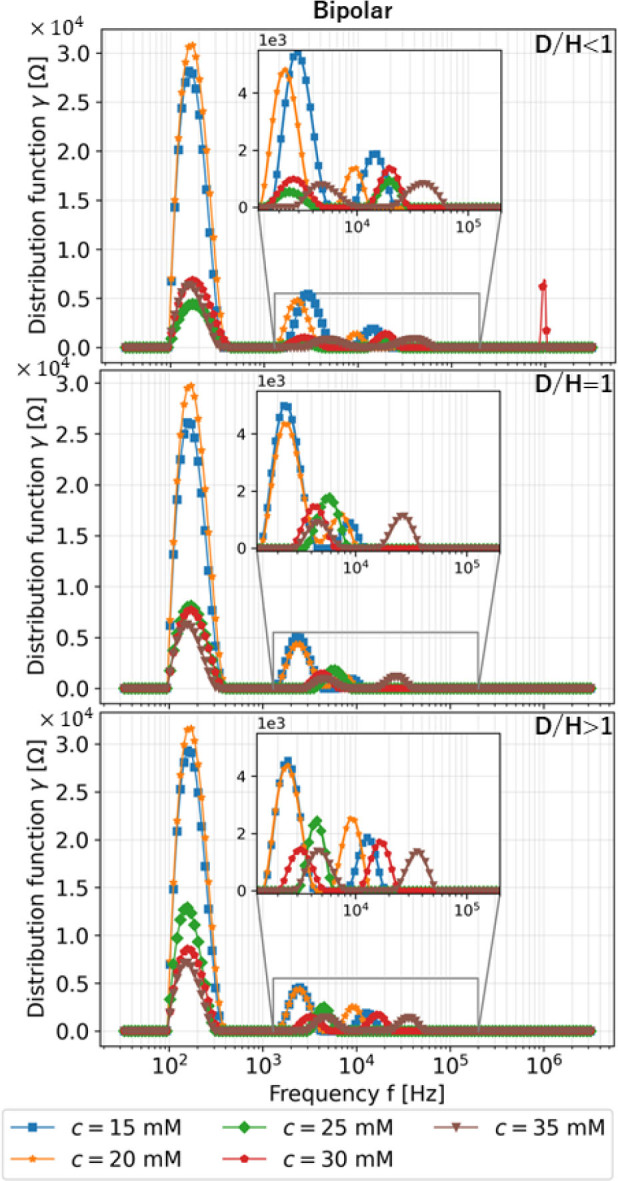
DRT results based on experiment with *φ_s_* = Bipolar in all frequencies.

**Fig. 9:**
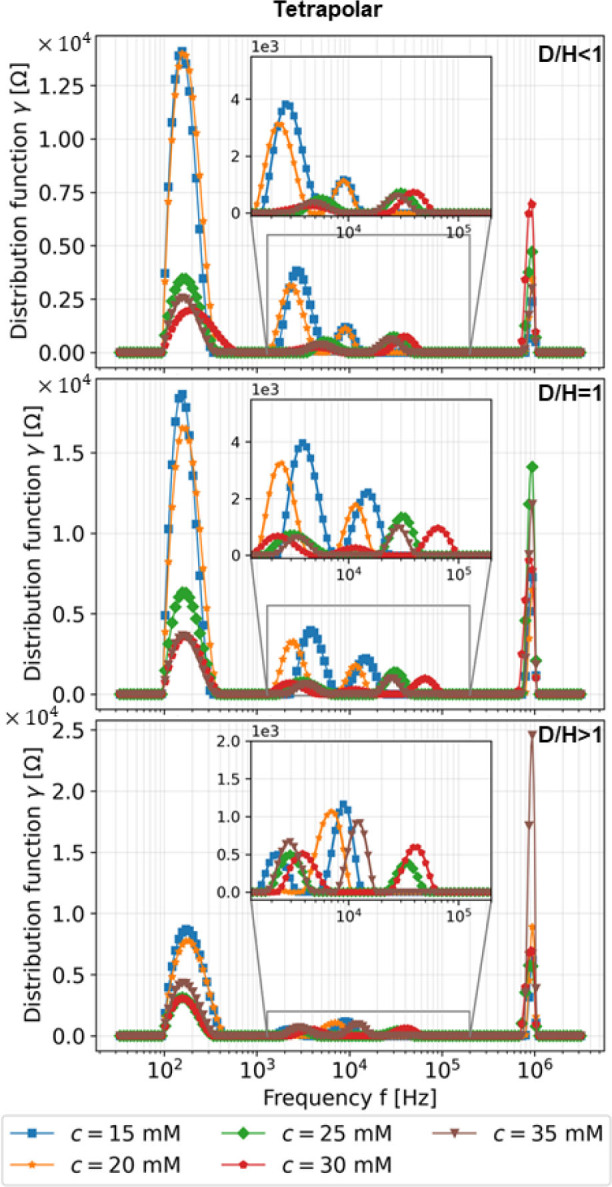
DRT results based on experiment with *φ_s_* = Tetrapolar in all frequencies.

Based on the results from DRT, **Fig. 8** and **Fig. 9** show the distribution function *γ* in all frequencies under various concentrations and all electrode-length-to-skin-thickness ratios for bipolar and tetrapolar mode, respectively, for the experimental studies. Different distribution function values of several local maximums also represent different conductivity change values from different concentrations, which means the system is indistinguishable and requires further investigation. Several local maximums are observed in both figures representing optimum frequencies at which the system exhibits maximum sensitivity or the most informative response. Hence, it gives a range of frequencies of interest that could be the location to extract the informative response of the system, which can be used for further investigation, in this particular case, for classification.

At high-frequency ranges, information about the stratum corneum known to have high resistance requires additional investigation [25]. The distribution function in the medium frequency regions is relatively low, requiring further experiments and analysis regarding the epidermis layer. Finally, the local maxima are due to dermis layer effect at low-frequency ranges since the increased injected saline water concentration is related to the decreased local maxima [17]. The distribution of relaxation time in simulation and experiment shows a similar trend. Thus, the heuristic selection of frequency pair shown in **Table 3** is acceptable.

### Skin layer classification

**Fig. 10:**
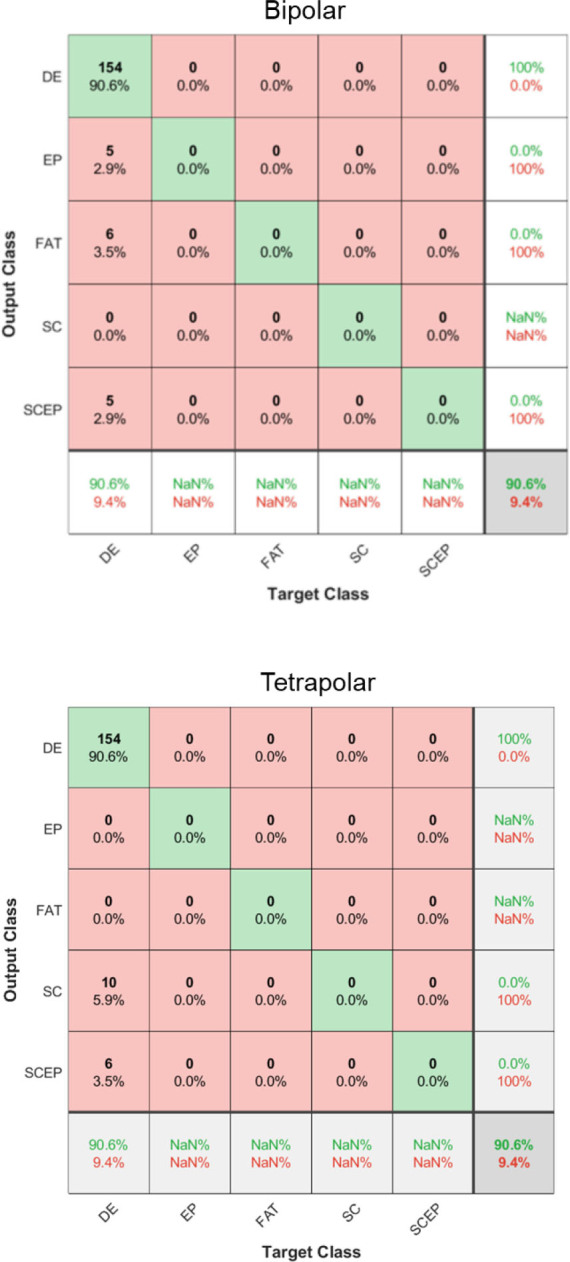
The confusion matrix shows the highest validation accuracy of experiments of porcine skin with FNN and four impedance inputs *α_ξ_*.

The confusion matrix is a useful tool for evaluating the performance of a classification model. In our studies, the FNN for bipolar mode at $f_{6}^{lh}$ achieved a high classification accuracy of 90.6% for the dermis layer (DE), which is promising. However, there were still some misclassifications, with 2.9% classified as epidermis (EP), 3.5% as fat (FAT), and 2.9% as stratum corneum and epidermis layers (SCEP). For tetrapolar mode at $f_{8}^{lh}$, the result showed that 90.6% of DE was classified correctly, while EP and FAT were classified at 0.0%, stratum corneum (SC) at 5.9%, and the SCEP at 3.5%, as shown in **Fig. 10**. The 0.0% classification in the confusion matrix does not indicate an error or confusion of DE for SC in bipolar or EP and FAT in tetrapolar injection patterns. Instead, it signifies that there were no instances of misclassification between these specific classes. Furthermore, the amount of data used in the analysis is sufficient for the purpose of this study. Also, it is important to note that the experimental conditions involved only the DE class for the model. While the overall accuracy is accountable, the misclassifications suggest room for improvement in the model.

One potential reason for the misclassifications could be the similarity between the dermis and epidermis layers in terms of their electrical properties [26]. Future studies could consider incorporating additional features [33] or imaging techniques [34], highlighting the importance of continuing to refine the model and improving the accuracy of the classification for these layers. Overall, the confusion matrix results demonstrate the potential of the FNN in accurately classifying skin layers but also highlight the need for further optimization and refinement of the model.

**Fig. 11:**
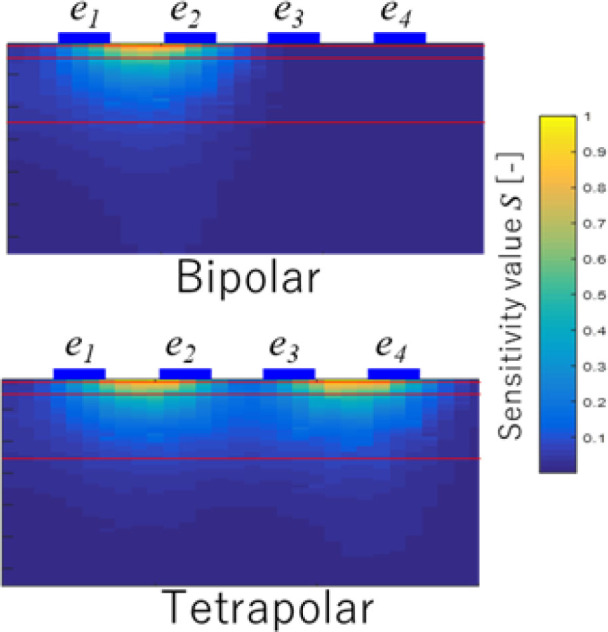
Comparison of sensitivity map distribution: bipolar and tetrapolar.

**Table 4** compares the FNN accuracy *Acc* between bipolar and tetrapolar injection patterns in each frequency pair variation. It shows that bipolar mode at $f_{6}^{lh}$ achieved the highest accuracy, *Acc* 90.6% and tetrapolar mode at $f_{8}^{lh}$ achieved the same accuracy. The study focused primarily on evaluating the classification performance and the influence of different injection patterns. It did not explicitly assess the effect of systematic error during the measurement. However, it is important to note that inherent systematic errors exist in the experimental results. Frequency pair is one of the ways to minimize this effect because systematic error due to stray capacitance or contact impedance is also frequency dependent. The discrepancy in performance between bipolar and tetrapolar injection patterns can be attributed to the difference in current pathway lengths. The variation in current pathway length directly affects the sensitivity of the measurement, as shown in **Fig. 11**. Therefore, the performances observed in **Table 4** result from these differences in current pathway length.

**Table 4:** Comparison of FNN accuracy *Acc* in terms of variation of frequency pair selection from experiment results.

Frequency pair $f_{r}^{lh}$ [kHz]	Bipolar *Acc* [%]	Tetrapolar *Acc* [%]
$f_{1}^{lh}=2\&10$	44.1	1.2
$f_{2}^{lh}=2\&35$	80.6	17.6
$f_{3}^{lh}=2\&100$	56.5	16.5
$f_{4}^{lh}=2\&225$	40.6	10.0
$f_{5}^{lh}=10\&35$	88.8	90.0
$f_{6}^{lh}=10\&100$	90.6	84.1
$f_{7}^{lh}=10\&225$	61.2	32.4
$f_{8}^{lh}=35\&100$	60.6	90.6
$f_{9}^{lh}=35\&225$	55.3	25.3
$f_{10}^{lh}=100\&225$	68.2	23.5

### Reliability of FNN

The study found that the FNN demonstrated promising results as a tool for skin layer classification, along with other bioelectrical impedance spectroscopy post-processing approaches.

Two injection patterns were employed to train the FNN, and while they performed differently, both had high accuracy. In order to validate these discrepancies, the sensitivity matrix map was used, which showed the effective area that passed by the electrical current distribution during the impedance measurement, as shown in **Fig. 11**. Although the sensitivity matrix map could differ from actual skin layer circumstances, it gave insight into the penetration depth of the electrical current under ideal conditions [35].

Real scenarios often involve an unknown skin thickness, making it difficult to evaluate the penetration depth of the electrical current by adjusting the electrode length. The study tested three different electrode-to-skin thickness ratios (D/H): D/H < 1, D/H = 1, and D/H > 1, corresponding to -10% to +50% of the skin thickness range. Under these conditions, results showed that the FNN had high accuracy rates in classifying skin layers in simulations and experiments. However, it could be challenging to obtain accurate results with electrode lengths similar to those of common biopotential electrodes like ECG (5 mm) or electromyography (10 mm). Further studies are necessary to investigate this issue.

### Reliability of training data features

The impedance inputs *α_ξ_*, proposed in this study, shows the reliability of features extraction to classify the conductivity change in the BIS measurement for skin layer classification. Using the impedance inputs *α_ξ_* in the FNN training reduces the number of training data significantly.

The conductivity change of skin layer affects all the impedance measurement parameters, which are impedance, phase angle, resistance, and reactance. By evaluating the results of **Fig. 4**, the impedance inputs *α_ξ_* in equations (4) to (7) are representations of a ratio metric form that amplifies the presence of conductivity change. As a result, the impedance inputs *α_ξ_* reduce the ill-posed problem of impedance measurement for skin layer classification of conductivity change.

In the experiment, measurement noise and systematic error inherently appear and are difficult to consider in the simulation. Nonetheless, the proposed method was confirmed by the experiment with skin because the data featuring based on impedance inputs *α_ξ_* at the selected frequency pair successfully eliminates the conductivity change that are not from the skin layer.

## Conclusions

In this study, the three objectives have been proposed for skin layer classification of conductivity change in skin layer, which are (1) to predict the validation accuracy of dermis classification by implementing feedforward neural network (FNN), (2) to minimize the unnecessary training data of FNN using four impedance inputs *α_ξ_*, which are the magnitude input *α*_|*Z*|_, phase angle input *α_θ_*, resistance input *α_R_*, and reactance input *α_X_*, and (3) to select the frequency pair $f_{r}^{lh}$.

From the simulation and experimental validation, some concluding remarks are as follows:

1. The trained FNN shows the highest accuracy *Acc* for bipolar and tetrapolar are 97.25% at $f_{2}^{lh}$ and 95.88% at $f_{10}^{lh}$, respectively, in the case of simulation. The experiment for bipolar has the highest accuracy, *Acc* = 90.6% at $f_{6}^{lh}$ and tetrapolar has *Acc* = 90.6% at $f_{8}^{lh}$.
2. The impedance inputs *α_ξ_* reduce the number of training data but has reliable data featuring FNN training by showing a high accuracy. All four proposed impedance inputs *α_ξ_* should be used to obtain a highest *Acc* for all injection patterns.
3. The frequency pair selection is satisfactory as bipolar mode has the highest validation accuracy *Acc* at $f_{6}^{lh}$, while tetrapolar mode has the highest accuracy *Acc* at $f_{8}^{lh}$.

## Appendix A

**Table 5:** Comparison studies of skin condition detection using only BIS or combined with machine learning algorithm.

No	Author	Skin Condition	Machine Learning Algorithm & Data Features	Frequency Pair Selection & Reason	Injection Patterns
1	Stig Ollmar [18]	Oral mucosa	*NA* & *Ź*, $\overset{´}{\Theta }$, *Ŕ*, and $X^{\prime }$	20 [kHz] & 50 [kHz]: *NA*	Bipolar
2	Nicander et al. [7]	Irritant dermatitis	*NA* & *Ź*	20 [kHz] & 1 [MHz]: *NA*	Bipolar
3	Ramos & Bertemes-Filho [36]	Skin cancer	*NA* & Electrical equivalent circuit	100 [Hz] – 1 [MHz]: To analyze the tetrapolar probe’s sensitivities’ frequency response. It was discovered that frequency has little effect on sensitivity.	Tetrapolar
4	Ferreira et al. [37]	Skin irritation	*NA* & Resistance ratio from two depth locations	20 [kHz] & 50 [kHz]: The ratio of total skin impedance obtained at low (20 [kHz]) and high frequencies (500 [kHz]) is the basis of the irritation indices.	Bipolar
5	Gessert et al. [12]	Skin melanoma	CNN and SVM & *Ź* and Images	1 [kHz] − 2.5 [MHz]: Following the device’s frequency range.	Bipolar
6	Luo et al. [38]	Skin cancer	*NA* & Transversal and longitudinal of relative permittivity and conductivity	8 – 256 [kHz]: For measurements of lesional and normal skin, intraclass correlation coefficient (ICC) conductivity values were low at 8 and 16 [kHz]. In contrast, relative permittivity ICC results displayed excellent repeatability at 16 [kHz].	Tetrapolar
7	Sarac et al. [39]	Skin cancer	*NA* & EIS Score	1 [kHz] – 2.5 [MHz]: The extracellular environment affects resistance to low frequencies, whereas both the intracellular and extracellular environments influence readings at higher frequencies.	Bipolar
8	This study	Skin layer classification	FNN & *α*_|*Z*|_, *α_θ_*, *α_R_*, and *α_X_*	$f_{r}^{lh}$ are selected based on DRT results.	Bipolar, Tetrapolar

1 Abbreviations: CNN, convolutional neural network; SVM, support vector machine; FNN. feedforward neural network.
